# Work ethics and general work attitudes in adolescents are related to quality of life, sense of coherence and subjective health – a Swedish questionnaire study

**DOI:** 10.1186/1471-2458-5-103

**Published:** 2005-10-07

**Authors:** Lars Axelsson, Ingemar Andersson, Anders Håkansson, Göran Ejlertsson

**Affiliations:** 1 Department of Health Sciences, Kristianstad University, S-291 88 Kristianstad, Sweden; 2 Department of Clinical Sciences, Malmö, Family Medicine, Lund University, S-205 02 Malmö, Sweden

## Abstract

**Background:**

Working life is an important arena in most people's lives, and the working line concept is important for the development of welfare in a society. For young people, the period before permanent establishment in working life has become longer during the last two decades. Knowledge about attitudes towards work can help us to understand young people's transition to the labour market. Adolescents are the future workforce, so it seems especially important to notice their attitudes towards work, including attitudes towards the welfare system. The aim of this study was to describe and analyse upper secondary school students' work attitudes, and to explore factors related to these attitudes.

**Methods:**

The sample consisted of 606 upper secondary school students. They all received a questionnaire including questions about quality of life (QOL), sense of coherence (SOC), subjective health and attitudes towards work. The response rate was 91%. A factor analysis established two dimensions of work attitudes. Multivariate analyses were carried out by means of logistic regression models.

**Results:**

Work ethics (WE) and general work attitudes (GWA) were found to be two separate dimensions of attitudes towards work. Concerning WE the picture was similar regardless of gender or study programme. Males in theoretical programmes appeared to have more unfavourable GWA than others. Multivariate analyses revealed that good QOL, high SOC and good health were significantly related to positive WE, and high SOC was positively related to GWA. Being female was positively connected to WE and GWA, while studying on a practical programme was positively related to GWA only. Among those who received good parental support, GWA seemed more favourable.

**Conclusion:**

Assuming that attitudes towards work are important to the working line concept, this study points out positive factors of importance for the future welfare of the society. Individual factors such as female gender, good QOL, high SOC and good health as well as support from both parents, positive experience of school and work contacts related positively to attitudes towards work. Further planning and supportive work have to take these factors into account.

## Background

The increasing number of people being outside the labour market in most European countries is a huge problem for both the societies and the individuals involved. Working life is an important arena in most people's lives, and also for a well-functioning society, so it is important to focus on people's attitudes towards work.

There are new demands and expectations of both the labour market and the workforce [1], and young people do not fully comply with these demands of improved flexibility [2]. According to a previous study, young people find it more acceptable to take advantage of social benefits compared to old people [3]. The structure of the labour market and working life is continuously changing, a development that has been especially obvious in recent decades. The working field has changed from a focus on industrial production towards a focus on information and media and improved educational skills [4]. Concerning young people, the period before permanent establishment in working life has become longer during the last two decades [5].

Working life is of fundamental importance for the development of welfare in a society [6]. An essential part of the welfare system is the working line concept [7], which has different meanings in different countries. The fundamental meaning of the concept is that everybody should earn his or her own living through work if possible. When needed, support to manage work should be given before public financial support is offered. Those who are unable to work should be financially compensated by the social welfare system. In Sweden, a debate is in progress about a general decline in ethics in the use of the social welfare system. This is probably related to huge restructuring of the public welfare system and increased individualisation among members of society [8].

The welfare system in Sweden, as in other parts of Scandinavia, is quite generous compared to many other countries, but the development of welfare also depends on the working line concept, which seems to bolster the welfare in a society. Living in generous welfare state like those in Scandinavia improves the experience of intrinsic work values rather than affecting work ethics negatively [9]. In Sweden, the social welfare compensation system is related to the loss of income through unemployment, disease and parental leave. However, the working line concept in Sweden has become weaker in its capability to motivate those on sick leave and in unemployment to re-enter working life [6].

Because important parts of the welfare system, and thereby equity between people in society, rely on the existence of a functional working line concept, it seems urgent to maintain it. Knowledge about attitudes towards work can help us to understand young people's transition into the labour market. As adolescents will constitute the forthcoming manpower, it seems especially important to notice their attitudes towards work, working life and the welfare system. In this respect, it has been highlighted that there is an extensive literature on personality and work, but the research on individual differences in attitudes and motivations to work seem to be rare [10].

Bearing in mind the great importance of the working line concept for a society's welfare, the aim of this study was to describe and analyse upper secondary school students' work attitudes, and to explore factors related to these attitudes.

## Methods

The Swedish upper secondary school consists of 16 different study programmes. The two theoretical programmes are either natural sciences or social sciences/humanities. The other 14 programmes are practical, training pupils for occupations, e.g. mechanics, building workers, industrial workers, electricians, assistant nurses or hotel and restaurant staff. Approximately 98% of all teenagers in Sweden attend upper secondary school, which lasts for three years.

This study was performed in Kristianstad municipality in the south of Sweden with approximately 75,000 inhabitants and with six upper secondary schools. About 1100 students were studying in their last year in those schools and about half of them were invited to participate in the study.

In January and February 2004, a questionnaire was distributed to 606 students (median age 18 years). In order to obtain a manageable and representative sample concerning sex and study programme, all students in three of the schools and all students in the theoretical programmes in another school were invited to participate in the study. In this study, students from 13 of the 16 ordinary programmes participated.

The questionnaire included questions about immigration, parental employment status, social support, subjective health and quality of life (QOL), satisfaction with aspects of life, sense of coherence (SOC), and different aspects of attitudes towards life and work. First, interviews, as a basis for the questions were performed with eleven students in practical and theoretical programmes. The questionnaire was developed, then tested in a pilot study among 14 students in practical and theoretical programmes, and adjusted before use.

The students answered the questionnaire anonymously in school. Students not present at the survey day received an envelope with the questionnaire when they were back in school again. Their main teacher collected the envelopes and returned them by post to the investigator. The final response rate was 91%, which means that 551 students responded to the questionnaire (Table 1).

**Table 1:** Number of students and percentages of dropout in theoretical and practical programmes. Males and females.

	**Theoretical programmes**		**Practical programmes**		
	Males	Females	Males	Females	Total

Number of students in the sample	103	128	245	130	606
Number of respondents	99	118	220	110	547^a^
Number of dropouts (%)	4 (4)	10 (8)	25 (10)	20 (15)	59 (9)

Note: ^a^Four respondents had not declared sex.

### Measurements and definitions

By using factor analysis, a set of answers to seven questions concerning attitudes towards work was analysed. Two dimensions of attitudes towards work appeared. The first four statements in Table 2 describe the attitudes towards parts of the social welfare system, i.e. unemployment and sick leave, and are labelled as work ethics (WE). The last three statements describe the general work attitudes (GWA) as a context, i.e. the importance of having a good life, having fun, and having spare time in relation to having a job.

**Table 2:** Results from the factor analysis of seven items from the questionnaire.

	**WE (Factor 1)**	**GWA (Factor 2)**
	Eigenvalue = 2.87	Eigenvalue = 1.09

	Factor loadings	Factor loadings

It is important that we all get a job, otherwise society will not function	-0.70	<0.10
It is OK to be unemployed even when there is a job available	0.78	0.18
It is OK to be reported sick even when not sick	0.60	0.28
It is unnecessary to work because the state provides compensation to the unemployed	0.63	0.10
		
It is possible to have a good life even without having a job	0.38	0.64
It is more important to have fun during life than to have a job	0.23	0.80
Spare time is more important for a good life than it is to have a job	<0.10	0.85

The response alternatives to each item were on a five-graded scale ranging from "completely agree" to "completely disagree". In order to perform logistic regression models, the dependent variables WE and GWA had to be dichotomised. A dichotomous WE variable was then constructed. Based on the logical distribution of the answers, those 69% who partly disagreed or completely disagreed with all four statements (the answers to the first statement were inverted before the WE variable was created) were designated as those who have *positive WE*. Those 31% who did not dismiss all the statements were designated as those having *negative WE*.

Another approach was used with the GWA factor because of the skewed distribution of answers. Those who partly disagreed or completely disagreed with all the three statements in the GWA variable were as few as 16%, so a GWA index ranging from 3–15 was made. The index was dichotomised as close as possible to the median; value 10–15 (high index) and value 3–9 (low index). High index means that the respondents disagreed rather than agreed with the statements and low index that they agreed rather than disagreed with the statements. The internal consistency of the index established by Cronbach's α was 0.71.

QOL, as we measured it, refers to the individuals' evaluation of their life contents, i.e. their global QOL. This definition is in accordance with definitions in earlier studies of QOL [11,12]. Global QOL is measured here by the answer to the question "How do you feel about your life as a whole just now?" This question had five response categories, scored from "very good" to "very bad". The item used in the questionnaire to measure QOL have been used previously [13]. The question has been chosen after pilot studies as an item giving meaningful results, and has been followed by interviews that confirm the results. This indicates that the questions asked have high validity when used in order to shed light on a person's global QOL [13].

Sense of coherence (SOC) was measured by Antonovsky's [14] Orientation to Life Questionnaire, short form (SOC-13) which includes 13 items with seven response alternatives each. This index ranged from 13–91 and was divided into four groups by quartiles. The internal consistency of the SOC index established by Cronbach's α was 0.85 in this study.

Subjective health was measured by the answers to the question: "How would you describe your overall health status at present?" This question had five response categories, scored from "very good" to "very bad". This question is in accordance with questions about self-rated health status in other studies i.e. [15].

As far as the other independent variables are concerned, the scales and the method for categorising the variables can be seen in Table 3.

**Table 3:** Independent variables in the logistic regression models.

**Variable**	**Type of data ^a^**	**Categorised**
Sex	N: Man/woman	Man/woman
Study programme	N: Theoretical/practical	Theoretical programmes/practical programmes
Work experiences during the whole upper secondary school period	O: Very positive (1) to very negative (5)	Very positive (1)/rather positive (2)/neither positive nor negative (3)/negative (4–5)
Contentedness with the upper secondary school period	O: Very content (1) to very discontented (5)	Content (1–2)/neither content nor discontented (3)/discontented (4–5)
Spare time	O: Very content (1) to very discontented (5)	Very content (1)/not very content (2–5)
Financial situation	O: Very content (1) to very discontented (5)	Content (1–2)/not content (3–5)
Parental support measured by two variables: support from father and support from mother	O: Very good support (1) to no support (5) and not relevant (6)	Very good support from both parents/very good support from one of them/not very good support from either of them (a combination of both variables established the three categories above)
Support from a friend	O: Very good (1) to no support (5) and not relevant (6)	Very good (1)/not very good support (2–6)
Possibilities to make own decisions about the future	O: Very good (1) to very bad (5)	Very good (1)/not very good (2–5)
QOL	O: Very good (1) to very bad (5)	Good (1–2)/not very good (3–5)
SOC index	Num: 13–91 points	Group 1 (25–50)/group 2 (51–59)/group 3 (60–68)/group 4 (69–91)
Subjective health	O: Very good (1) to very bad (5)	Good (1–2)/not very good (3–5)

^a ^N = Nominal; O = Ordinal; Num = Numerical.

### Statistical analyses

The significance of bivariate relations between variables was tested by chi-squared test. When the groups were small or the expected frequencies were low, Fisher's exact test was used for the comparison. The significance of differences between means was tested by a one-way Anova test. Correlation was established by Spearman's rank order correlation coefficient.

The factor analysis was done by means of principal component analysis. For factor extraction the varimax method for orthogonal rotation was used and eigenvalue >1 was set as a criterion. As can be seen from Table 2, variables with practically significant (>0.5) varimax loadings [16] were used to establish logical units (WE and GWA).

Multivariate analyses were carried out as logistic regression models (method: enter). The WE variable and the dichotomised GWA index were used as dependent variables. Variables included in the model were those with a significant (p < 0.20) relation to the dependent variable and with low correlation (r_s_^2 ^< 0.20) to each other. As QOL, SOC and subjective health were highly correlated, they were analysed in separate models (Tables 6 and 7).

**Table 6:** Positive odds ratios (POR) and 95% confidence intervals (CI) for variables related to positive work ethics (WE), in three different models using QOL, SOC and subjective health as explanatory variables (n = 540).

	QOL included		SOC included		Subjective health included	
	POR	CI	POR	CI	POR	CI

Sex: female	**1.60**	**1.06–2.44**	**1.88**	**1.21–2.93**	**1.65**	**1.08–2.51**
Study programme: practical	1.22	0.81–1.84	1.23	0.80–1.89	1.17	0.78–1.77
Work experiences:						
negative	1.00		1.00		1.00	
neither/nor	1.11	0.46–2.72	1.04	0.42–2.56	1.16	0.48–2.82
rather positive	2.05	0.88–4.81	1.76	0.75–4.16	2.18	0.94–5.09
very positive	**3.60**	**1.46–8.85**	**3.13**	**1.26–7.80**	**3.89**	**1.59–9.50**
Spare time: very content	0.95	0.60–1.51	0.95	0.61–1.50	1.06	0.67–1.68
Support from a friend: very good	0.88	0.58–1.34	0.82	0.54–1.26	0.87	0.58–1.32
Possibilities to make own decisions about the future: very good	1.48	0.93–2.36	1.46	0.92–2.32	**1.63**	**1.03–2.56**
QOL:						
not good	1.00					
rather good	**1.98**	**1.24–3.15**				
very good	**3.01**	**1.58–5.75**				
SOC:						
group 1, 25–50			1.00			
group 2, 51–59			**1.73**	**1.02–2.94**		
group 3, 60–68			**2.24**	**1.26–3.96**		
group 4, 69–91			**4.25**	**2.21–8.16**		
Subjective health:						
not good					1.00	
good					**1.76**	**1.12–2.78**
very good					**2.06**	**1.12–3.79**

Notes: Figures in bold when POR significant.

Variables not showing a bivariate relation (p < 0.20) to WE and therefore not included in the logistic regression model were: immigration, parental employment status, contentedness with the upper secondary school period, support from a relative, partner, other persons, number of social contacts outside home, close contact with persons outside home, parental support, self-confidence, contentedness with the personal financial situation, relations to friends, feeling lonesome, length of work experience, plans for a working or studying career.

**Table 7:** Positive odds ratios (POR) and 95% confidence intervals (CI) for variables related to high general work attitude (GWA) index, in three different models using QOL, SOC and subjective health as explanatory variables (n = 514–525).

	**QOL included**		**SOC included**		**Subjective health included**	
	POR	CI	POR	CI	POR	CI

Sex: female	**2.38**	**1.61–3.51**	**2.77**	**1.84–4.16**	**2.40**	**1.62–3.54**
Study programme: practical	**1.81**	**1.23–2.66**	**1.81**	**1.22–2.69**	**1.80**	**1.22–2.65**
Spare time: very content	1.06	0.71–1.60	0.95	0.63–1.44	1.07	0.71–1.60
Financial situation: content	1.41	0.96–2.08	1.27	0.85–1.90	1.43	0.97–2.10
Support from a friend: very good	0.74	0.50–1.10	0.69	0.46–1.04	0.74	0.50–1.10
Contentedness with the upper secondary school period:						
discontent	1.00		1.00		1.00	
neither/nor	1.73	0.81–3.72	1.78	0.83–3.81	1.71	0.79–3.68
content	**2.09**	**1.08–4.07**	**1.93**	**1.00–3.72**	**2.09**	**1.08–4.06**
Parental support:						
not very good	1.00		1.00		1.00	
very good support from father or mother	1.55	0.94–2.54	1.49	0.90–2.47	1.55	0.95–2.54
very good support from both parents	**1.66**	**1.04–2.65**	1.57	0.98–2.54	**1.66**	**1.04–2.65**
QOL: good	1.19	0.73–1.94				
SOC: high			**1.92**	**1.28–2.90**		
Subjective health:						
good					1.19	0.75–1.88

Notes: Figures in bold when POR significant.

Variables not showing a bivariate relation (p < 0.20) to GWA index and therefore not included in the logistic regression model were: immigration, parental employment status, close contact with persons outside home, support from a relative, support from other persons, relations to friends, number of social contacts outside home, support from partner, self-confidence, feeling lonesome, possibilities to make own decisions, length of work experience, work experiences, plans of a working or studying career.

Independent variables were first analysed as categorical, and when not significantly related to the dependent variable they were presented as dichotomous instead. They were dichotomised as close as possible to the median value in order to give a neutral split, free from subjective influence (Table 3). The goodness of fit for the logistic regression models used was established by Hosmer and Lemeshow's [17] goodness of fit test, i.e. a not significant goodness of fit test, and high agreement was found between observed and expected frequencies in the two by ten tables derived from these tests.

The results of the logistic regression models were expressed as odds ratios (OR) with 95% confidence interval (CI). The odds ratios were calculated in an ordinary way, but in line with the salutogenetic approach the positive and negative outcome in the dependent variable, as well as in the explanatory variables, were changed and expressed as positive odds ratios (POR) [18].

The significance level was set to 0.05. For the statistical procedures SPSS for Windows version 9.0 and EPI 5 (Epi Info) were used.

The study has been approved by the Ethics Committee at the Faculty of Medicine, University of Lund (LU 669-03).

## Results

The factor analysis established work ethics (WE) and general work attitudes (GWA) as two separate dimensions of attitudes towards work, and the correlation between those two components was r_s _= 0.32. As can be seen from Table 4, there were no differences in the four statements that constituted WE according to sex and study programme. Almost all of the students disagreed with the statements concerning sick leave and unemployment benefits. Concerning GWA, there were significant differences in the three statements principally because fewer males, especially in theoretical programmes, had disagreed with the statements. These differences can also be seen according to the GWA index (Table 5).

**Table 4:** Agreement/disagreement with the statements about work ethics (WE) and general work attitudes (GWA) and % in relation to gender and type of programme.

	**Theoretical programmes**		**Practical programmes**		
	Males	Females	Males	Females	p-value^a^

**WE**	n = 97–99	n = 115–118	n = 216–219	n = 108–110	

Agree that It is important that we all get a job otherwise the society doesn't work	80	85	89	89	0.115
Disagree that It is OK to be unemployed even when there is a job available	87	95	93	96	0.066
It is OK to be reported sick even when not sick	95	98	94	97	0.387
It is unnecessary to work because the state provides compensation to the unemployed	96	98	97	97	0.775
**GWA**					
Disagree that It is possible to have a good life even without having a job	57	75	72	81	**0.001**
It is more important to have fun during life than to have a job	60	73	73	85	**0.001**
Spare time is more important for a good life than it is to have a job	53	77	68	77	**0.000**

Notes: χ^2 ^analyses when comparing distributions of all four groups.

^a ^p-values in bold when significant.

**Table 5:** Work ethics (WE), general work attitudes (GWA), QOL, SOC and subjective health according to study programmes and sex.

	**Theoretical programmes**		**Practical programmes**		
	Males	Females	Males	Females	p-value^a^

	(n = 95–99)	(n = 114–118)	(n = 213–220)	(n = 107–110)	

Positive WE %	60	72	69	76	0.062
High GWA index %	30	56	47	63	**0.000**
Good QOL %	75	77	81	71	0.214
Good subjective health %	71	70	81	66	**0.008**
SOC: Mean	60.2	58.7	61.4	55.9	**0.003**

Notes: χ^2 ^analyses when comparing distributions of all four groups of WE, GWA-index, QOL and subjective health and a one-way Anova test for the comparison of all four groups of SOC.

^a ^p-values in bold when significant.

According to our definition of WE, 69% of the students reported positive WE. The highest percentages of students with good subjective health and the highest SOC scores were found among the males in practical programmes (Table 5).

The logistic regression model that includes QOL as an explanatory variable (Table 6) shows that positive WE was related to being female (POR = 1.60), having very positive work experiences (POR = 3.60), and having rather or very good QOL (POR = 1.98 and 3.01, respectively). SOC and subjective health correlated highly with each other and with QOL (r_s_^2 ^> 0.20), and were therefore not included in the same logistic regression model. When additional logistic regression models were tested with SOC or subjective health as explanatory variables instead of QOL, both SOC and subjective health were significantly related to WE. Principally the same variables, with almost the same figures of POR, were significantly related to WE when QOL, SOC or subjective health were used as explanatory variables (Table 6).

In another logistic regression model including QOL as an explanatory variable (Table 7), it is shown that high GWA index was related to being female (POR = 2.38), studying on a practical programme (POR = 1.81), being content with the upper secondary school period (POR = 2.09), and having very good parental support (POR = 1.66). In contrast to QOL and subjective health, SOC was significantly related to GWA. Otherwise using SOC, subjective health or QOL as explanatory variables gave similar results in relation to GWA.

## Discussion

Our study presents data on attitudes towards work among adolescents. Two independent factors, work ethic (WE) and a general work attitude (GWA) appeared as important.

According to our results, having negative WE and low GWA index include negative attitude towards work as an action of solidarity with society. A majority among the students reported positive WE. Focusing on factors that are related to positive WE and GWA might open ways to moderate young people's outlook concerning work in relation to the welfare system.

It can be assumed that negative WE and a low GWA index also mirror negative attitudes towards the working line concept. But the societal norm, on which the working line concept relies, is that as many as possible should earn their living through work if possible.

Consequently, it is important that citizens do not use benefits derived from the social welfare system except when necessary. Therefore, support to people, making them enter working life or encouraging them to stay in working life, is important. Support in this can be built up by knowledge about factors that are related to attitudes towards work. Our results in this study highlight some variables that are significantly related to attitudes towards work, in terms of WE and GWA.

First, having positive WE was related to good QOL, high SOC and good subjective health, and having high GWA index was related to high SOC. However, the result showed that the figures for POR when SOC or subjective health were used as explanatory variables in the models were principally the same as when QOL was used in the model. Antonovsky's [14] SOC concept has affinities with other salutogenetic concepts, e.g. self-efficacy [19] and hardiness [20]. Antonovsky [14] suggested that the stronger the SOC world outlook of a person is, the more likely he or she is to cope successfully with life stressors. He also suggested that SOC contributes to good health as an individual with high SOC will perceive stressors to be less stressful and the negative consequences of a stressful life would therefore be less. Recently it has been suggested that SOC has a unique relation to health and that the concept of SOC also refers to an active self-esteem structure and self-determination [21]. Based on our results, we suggest that QOL and subjective health, although measured as single items, may be markers of a similar concept to SOC, which is related to WE and GWA as well.

WE and GWA represent different dimensions of attitudes towards work, but the results derived from the logistic regression models also suggest similarities in the outcomes concerning these variables i.e. in gender and work or school experiences. Females reported a more positive WE and a higher GWA index than males did. One explanation from the different attitudes can be found in males' and females' different work values. It has been postulated that a radical change in attitudes and values concerning work is in progress [22]. "Postmateralistic" values such as the importance of good quality of life, high self-expression, belonging and intellectual satisfaction, are ranked higher, especially by females, than materialistic values such as economic growth, law and order and security. It has also been shown that females ranked altruism as a work value [23], and endorsed work ethics more than males did [10]. Our results show that males expressed a more positive attitude towards being voluntarily unemployed and to improper use of the social welfare system, and they gave less priority to work as something important for a good life compared to females.

In the present study, WE relates to former work experiences and GWA relates to former school experiences. For many students, work experiences occur during the upper secondary school period. It is also common that young people work part-time in the paid labour market in the evenings, and/or in the summer while still full-time students [24]. If these experiences of work are mainly positive it seems reasonable that they influence WE positively.

It has previously been shown that the quality of work experiences during high school has significant consequences for well-being among young people[25]. On the one hand, work can be beneficial for youth development because work can limit depressive affect. On the other hand, employment including early work stressors can cause harmful consequences to adolescents' mental well being [25]. It seems important that schools work to obtain high-quality practical placements for the students and that employers are aware of the connection between WE and work experiences. In order to moderate the experiences of work to give positive events, schools and employers can support adolescents in balancing their commitments in both school and work.

GWA was not related to former work experiences, but was related to whether the upper secondary school period is a positive event or not. In Sweden, 97% of adolescents apply to upper secondary school in their final year of compulsory school, although one of five lacks an upper secondary school education at the age of 20 [26]. The most common reason for leaving after compulsory school or for dropping out of upper secondary school was school fatigue. Our results show that those who were less content with the school period also had low GWA index. Therefore, school experiences, i.e. school fatigue, should be recognised, as they can influence willingness to complete school and thereby secondarily affect the possibilities to enter working life as well.

Parental support is an important part of social support, and in this study parental support was related to GWA, but not WE. Social support has been described as a moderator of life stress and as a potent isolator against stressful conditions in life [27]. It has also been shown that young people perceive family/friends as the primary providers of social support [28]. Concerning unemployed young people, it has recently been shown that parental support decreases the risk of having mental health problems [29]. It has also been suggested that fathers are important in supporting young people to enter working life [26]. Our result is complementary to that; very good support from both parents relates to the attitude that work is important for a good life, an attitude that can be assumed to increase the chances of entering working life.

According to our results, those who follow a practical study programme are more likely than those in theoretical programmes to have high GWA index. One explanation might be that those in practical programmes give more priority to working life, i.e. they are concentrated on getting a job when school is finished, as they have recently chosen and accomplished a work-related education. It can also be assumed that those in theoretical programmes, to a larger extent than those in practical programmes, give priority to further studies. It has been shown that students in work-related courses pay less importance to work goals than students in theoretical programmes do [30]. In the present study, high GWA index is related to studying on a practical programme. Therefore, it seems possible that those students pay more attention to working life than those in theoretical programmes do.

### Methodological considerations

The advantage of this study is that 606 students were invited to participate and most of them (91%) completed the study. A non-response analysis showed no significant differences in dropout according to sex or study programme. Thus, our results might be generalised to other students in similar settings. The result of the factor analysis makes sense. The appropriateness of the analyses was 0.80 when established by Kaiser-Meyer-Olkin measure of sampling adequacy. The factor analysis logically combined items, with practically significant factor loadings, that established work ethics and general work attitudes as two important components in students' attitudes towards work. The study design was cross-sectional, which limits the possibilities to draw causal conclusions. Therefore we refer to relations between variables instead of emphasising the concept determinants. Another limitation of the results is the possibility of bias related to the self-report. The questions asked should have limited recall problems but some statements of WE may give socially acceptable answers. In that way the figure of 69% reporting a positive WE could be some overestimation. However, the procedure with strictly anonymous responses may partly compensate for such bias. The ethical attitude could also be influenced by personality traits not covered in our study. The findings suggest that interpretations of the students' attitudes towards work, working life and the welfare system and related variables might be a basis for further studies on this field.

## Conclusion

Factors related to attitudes towards work are of importance for increased understanding of adolescents' transition to working life. Several factors appeared as positively related to either of the two dimensions of work attitudes found in this study: female gender, good QOL, high SOC, support from both parents, school experiences and work contacts. It is important that schools as well as employers help students to balance their study commitments and work as it might improve experiences of school and work. Knowledge about these factors might increase adolescents' possibilities to enter working life. However, further studies of this kind that explore attitudes towards work among adolescents are necessary for an understanding of young adults' transition to working life.

## Competing interests

The author(s) declare that they have no competing interests.

## Authors' contributions

LA participated in conceiving the study, carrying out the study and had the main responsibility for writing the manuscript.

IA participated in conceiving the study and writing the manuscript.

AH participated in conceiving the study and writing the manuscript.

GE participated in conceiving the study and writing the manuscript.

All authors read and approved the final manuscript.

## Pre-publication history

The pre-publication history for this paper can be accessed here:


